# 2,4-Diamino-6-methyl-1,3,5-triazin-1-ium chloride

**DOI:** 10.1107/S1600536810007907

**Published:** 2010-03-06

**Authors:** Hui-Fen Qian, Wei Huang

**Affiliations:** a College of Sciences, Nanjing University of Technology, Nanjing 210009, People’s Republic of China; b State Key Laboratory of Coordination Chemistry, Nanjing National Laboratory of Microstructures, School of Chemistry and Chemical Engineering, Nanjing University, Nanjing 210093, People’s Republic of China

## Abstract

In the title compound, C_4_H_8_N_5_^+^·Cl^−^, a two-dimensional layer packing network is observed in which every chloride anion links three adjacent 2,4-diamino-6-methyl-1,3,5-triazin-1-ium cations by N—H⋯Cl hydrogen-bonding inter­actions, forming 12-membered and eight-membered hydrogen-bonded rings with graph-set motifs *R*_4_^4^(12) and *R*_3_^3^(8), respectively. In addition, N—H⋯N hydrogen bonds are found between adjacent cations, forming another type of eight-membered [*R*_2_^2^(8)] hydrogen-bonded ring.

## Related literature

For related complexes, see Delori *et al.* (2008 ▶); Fan *et al.* (2009 ▶); Perpétuo & Janczak (2007 ▶); Portalone & Colapietro (2007 ▶); Wijaya *et al.* (2004 ▶).
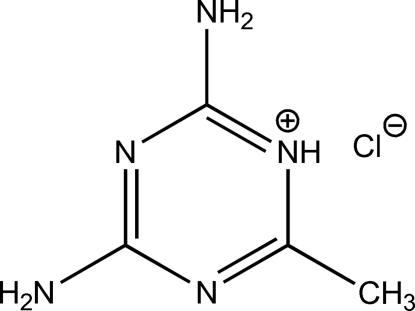


## Experimental

### 

#### Crystal data

C_4_H_8_N_5_^+^·Cl^−^
*M* *_r_* = 161.60
Triclinic, 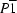

*a* = 5.6449 (11) Å
*b* = 7.8723 (15) Å
*c* = 9.3476 (17) Å
α = 65.551 (3)°
β = 75.779 (2)°
γ = 71.027 (2)°
*V* = 354.61 (12) Å^3^
*Z* = 2
Mo *K*α radiation
μ = 0.47 mm^−1^
*T* = 291 K
0.16 × 0.14 × 0.10 mm

#### Data collection

Bruker SMART 1K CCD area-detector diffractometer
Absorption correction: multi-scan (*SADABS*; Sheldrick, 1996 ▶) *T*_min_ = 0.929, *T*_max_ = 0.955
1871 measured reflections
1303 independent reflections
1042 reflections with *I* > 2σ(*I*)
*R*_int_ = 0.082

#### Refinement

*R*[*F* ^2^ > 2σ(*F* ^2^)] = 0.039
*wR*(*F* ^2^) = 0.111
*S* = 1.07
1303 reflections
96 parameters
2 restraints
H atoms treated by a mixture of independent and constrained refinement
Δρ_max_ = 0.25 e Å^−3^
Δρ_min_ = −0.28 e Å^−3^

### 

Data collection: *SMART* (Bruker, 2007 ▶); cell refinement: *SAINT* (Bruker, 2007 ▶); data reduction: *SAINT*; program(s) used to solve structure: *SHELXTL* (Sheldrick, 2008 ▶); program(s) used to refine structure: *SHELXTL*; molecular graphics: *SHELXTL*; software used to prepare material for publication: *SHELXTL*.

## Supplementary Material

Crystal structure: contains datablocks global, I. DOI: 10.1107/S1600536810007907/nk2023sup1.cif

Structure factors: contains datablocks I. DOI: 10.1107/S1600536810007907/nk2023Isup2.hkl

Additional supplementary materials:  crystallographic information; 3D view; checkCIF report

## Figures and Tables

**Table 1:** Hydrogen-bond geometry (Å, °)

*D*—H⋯*A*	*D*—H	H⋯*A*	*D*⋯*A*	*D*—H⋯*A*
N3—H3⋯Cl1^i^	0.86 (1)	2.25 (1)	3.107 (2)	174 (3)
N4—H4*D*⋯Cl1^ii^	0.86	2.52	3.372 (2)	169
N4—H4*E*⋯N1^iii^	0.86	2.32	3.171 (3)	170
N5—H5*A*⋯N2^ii^	0.86	2.15	3.008 (3)	174
N5—H5*B*⋯Cl1	0.86	2.40	3.125 (2)	143

Symmetry codes: (i) 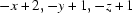; (ii) 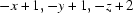; (iii) 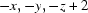.

**Fig. 1.**
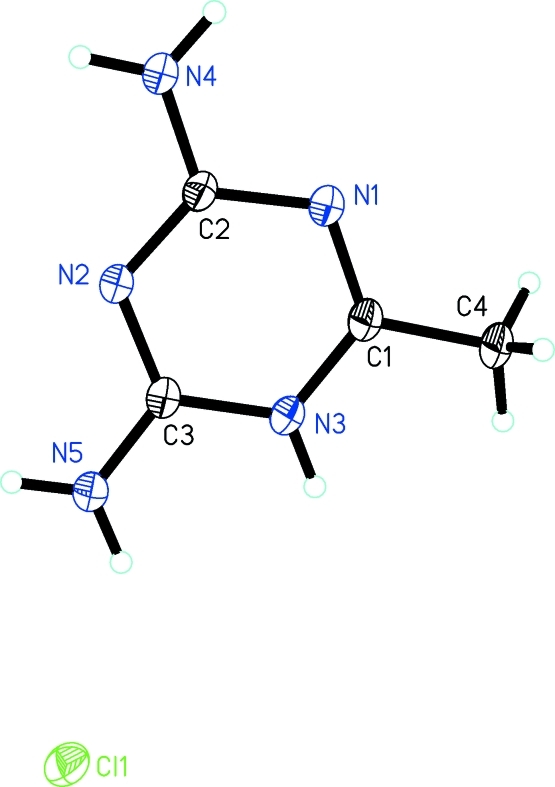
The molecular structure of the title compound with the atom-numbering scheme. Displacement ellipsoids are drawn at the 30% probability level.

**Fig. 2.**
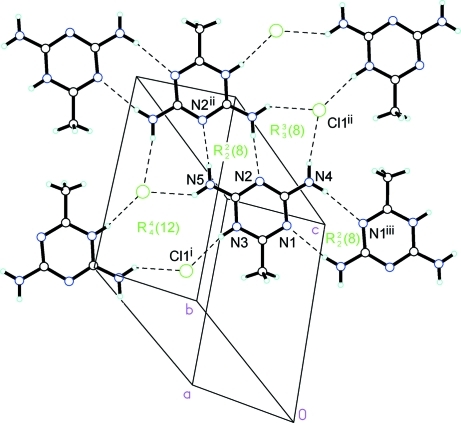
Perspective view of the hydrogen bonding interactions within one layer in (I), where the hydrogen bonds are shown as dashed lines. [Symmetry codes: (i) -x+2, -y+1, -z+1; (ii) -x+1, -y+1, -z+2, (iii) -x, -y, -z+2.]

## supplementary crystallographic information

### Comment

To date, a series of clathrates and acid-base adducts of 2,4-diamino-6-methyl-1,3,5-triazine have been structurally reported (Delori *et al.*, 2008; Fan *et al.*, 2009; Perpétuo *et al.*, 2007; Portalone *et al.*, 2007; Wijaya *et al.*, 2004). In this paper, we report the X-ray single-crystal structure of 2,4-diamino-6-methyl-1,3,5-triazin-1-ium chloride (I).

The molecular structure of (I) is illustrated in Fig. 1. The mean deviation from a least-squares plane for all the non-hydrogen atoms of the cations is 0.0039 (1) Å, while that for all the non-hydrogen atoms of (I) including the chloride anion is 0.0041 (1) Å. It is interesting to note that every chloride anion links three adjacent 2,4-diamino-6-methyl-1,3,5-triazin-1-ium cations by N—H···Cl hydrogen bonding interactions forming two kinds of twelve-membered [*R*_4_^4^(12)] and eight-membered [*R*_3_^3^(8)] hydrogen-bonded rings. In addition, N—H···N hydrogen bonding interactions are found between nitrogen atoms N1, N4 and N2, N5 from neighbouring cations, respectively, forming another type of eight-membered [*R*_2_^2^(8)] hydrogen-bonded rings. With the help of above-mentioned N—H···N and N—H···Cl hydrogen bonds, a two-dimensional layer packing network is finally constituted (Fig. 2).

### Experimental

The title compound was purchased directly from Kangmanlin Co. in China and the colourless single crystals of (I) suitable for X-ray diffraction determination were obtained from a mixture of water and ethanol in a ration of 1:3 (v/v) by slow evaporation at room temperature in air for one week.

### Refinement

The H atoms bonded to carbon atoms were placed in geometrically idealized positions and refined as riding with C—H = 0.96 Å and *U*_iso_(H) = 1.5*U*_eq_(C). The H atom bonded to nitrogen atom was located in the difference synthesis and were refined isotropically.

### Crystal data

**Table:**

C_4_H_8_N_5_^+^·Cl^−^	*Z* = 2
*M**_r_* = 161.60	*F*(000) = 168
Triclinic, *P*1	*D*_x_ = 1.513 Mg m^−^^3^
Hall symbol: -P 1	Mo *K*α radiation, λ = 0.71073 Å
*a* = 5.6449 (11) Å	Cell parameters from 890 reflections
*b* = 7.8723 (15) Å	θ = 2.4–28.0°
*c* = 9.3476 (17) Å	µ = 0.47 mm^−^^1^
α = 65.551 (3)°	*T* = 291 K
β = 75.779 (2)°	Block, colourless
γ = 71.027 (2)°	0.16 × 0.14 × 0.10 mm
*V* = 354.61 (12) Å^3^	

### Data collection

**Table:**

Bruker SMART 1K CCD area-detector diffractometer	1303 independent reflections
Radiation source: sealed tube	1042 reflections with *I* > 2σ(*I*)
graphite	*R*_int_ = 0.082
ω scans	θ_max_ = 25.5°, θ_min_ = 2.4°
Absorption correction: multi-scan (*SADABS*; Sheldrick, 1996)	*h* = −6→6
*T*_min_ = 0.929, *T*_max_ = 0.955	*k* = −9→8
1871 measured reflections	*l* = −11→11

### Refinement

**Table:**

Refinement on *F*^2^	Primary atom site location: structure-invariant direct methods
Least-squares matrix: full	Secondary atom site location: difference Fourier map
*R*[*F*^2^ > 2σ(*F*^2^)] = 0.039	Hydrogen site location: difference Fourier map
*wR*(*F*^2^) = 0.111	H atoms treated by a mixture of independent and constrained refinement
*S* = 1.07	*w* = 1/[σ^2^(*F*_o_^2^) + (0.0566*P*)^2^] where *P* = (*F*_o_^2^ + 2*F*_c_^2^)/3
1303 reflections	(Δ/σ)_max_ < 0.001
96 parameters	Δρ_max_ = 0.25 e Å^−^^3^
2 restraints	Δρ_min_ = −0.28 e Å^−^^3^

### Special details

**Table:**

Experimental. The structure was solved by direct methods (Bruker, 2007) and successive difference Fourier syntheses.
Geometry. All esds (except the esd in the dihedral angle between two l.s. planes) are estimated using the full covariance matrix. The cell esds are taken into account individually in the estimation of esds in distances, angles and torsion angles; correlations between esds in cell parameters are only used when they are defined by crystal symmetry. An approximate (isotropic) treatment of cell esds is used for estimating esds involving l.s. planes.
Refinement. Refinement of *F*^2^ against ALL reflections. The weighted *R*-factor wR and goodness of fit *S* are based on *F*^2^, conventional *R*-factors *R* are based on *F*, with *F* set to zero for negative *F*^2^. The threshold expression of *F*^2^ > σ(*F*^2^) is used only for calculating *R*-factors(gt) etc. and is not relevant to the choice of reflections for refinement. *R*-factors based on *F*^2^ are statistically about twice as large as those based on *F*, and *R*- factors based on ALL data will be even larger.

### Fractional atomic coordinates and isotropic or equivalent isotropic displacement parameters (Å^2^)

**Table:**

	*x*	*y*	*z*	*U*_iso_*/*U*_eq_	
C1	0.4096 (5)	0.1560 (3)	0.7585 (3)	0.0347 (6)	
C2	0.2148 (4)	0.2118 (3)	0.9841 (3)	0.0321 (5)	
C3	0.5223 (4)	0.3642 (3)	0.8366 (3)	0.0328 (6)	
C4	0.4508 (5)	0.0666 (4)	0.6414 (3)	0.0458 (7)	
H4A	0.4399	−0.0647	0.6949	0.069*	
H4B	0.6152	0.0696	0.5817	0.069*	
H4C	0.3244	0.1365	0.5709	0.069*	
Cl1	1.09342 (12)	0.67148 (10)	0.58725 (8)	0.0492 (3)	
N1	0.2385 (4)	0.1200 (3)	0.8830 (2)	0.0375 (5)	
N2	0.3507 (4)	0.3320 (3)	0.9661 (2)	0.0338 (5)	
N3	0.5556 (4)	0.2746 (3)	0.7331 (2)	0.0351 (5)	
N4	0.0432 (4)	0.1740 (3)	1.1080 (2)	0.0433 (6)	
H4D	0.0189	0.2262	1.1767	0.052*	
H4E	−0.0452	0.0969	1.1205	0.052*	
N5	0.6634 (4)	0.4803 (3)	0.8085 (2)	0.0443 (6)	
H5A	0.6466	0.5365	0.8733	0.053*	
H5B	0.7731	0.5003	0.7252	0.053*	
H3	0.654 (4)	0.297 (4)	0.6445 (19)	0.058 (9)*	

### Atomic displacement parameters (Å^2^)

**Table:**

	*U*^11^	*U*^22^	*U*^33^	*U*^12^	*U*^13^	*U*^23^
C1	0.0390 (14)	0.0376 (13)	0.0335 (13)	−0.0099 (11)	0.0005 (10)	−0.0214 (10)
C2	0.0379 (13)	0.0339 (12)	0.0281 (12)	−0.0104 (10)	0.0016 (10)	−0.0170 (10)
C3	0.0378 (13)	0.0349 (12)	0.0309 (12)	−0.0106 (10)	−0.0010 (10)	−0.0177 (10)
C4	0.0544 (17)	0.0604 (17)	0.0401 (14)	−0.0242 (14)	0.0072 (12)	−0.0352 (13)
Cl1	0.0474 (5)	0.0639 (5)	0.0421 (4)	−0.0230 (3)	0.0105 (3)	−0.0272 (3)
N1	0.0442 (13)	0.0421 (12)	0.0352 (12)	−0.0180 (10)	0.0051 (10)	−0.0232 (9)
N2	0.0400 (12)	0.0386 (11)	0.0303 (10)	−0.0161 (9)	0.0038 (9)	−0.0198 (9)
N3	0.0403 (12)	0.0403 (11)	0.0312 (11)	−0.0145 (10)	0.0053 (9)	−0.0216 (9)
N4	0.0524 (13)	0.0533 (13)	0.0392 (12)	−0.0276 (11)	0.0114 (10)	−0.0300 (10)
N5	0.0539 (14)	0.0555 (13)	0.0393 (12)	−0.0311 (11)	0.0121 (10)	−0.0294 (10)

### Geometric parameters (Å, °)

**Table:**

C1—N1	1.311 (3)	C4—H4A	0.9600
C1—N3	1.352 (3)	C4—H4B	0.9600
C1—C4	1.468 (3)	C4—H4C	0.9600
C2—N4	1.312 (3)	N3—H3	0.862 (11)
C2—N2	1.337 (3)	N4—H4D	0.8600
C2—N1	1.369 (3)	N4—H4E	0.8600
C3—N5	1.308 (3)	N5—H5A	0.8600
C3—N2	1.341 (3)	N5—H5B	0.8600
C3—N3	1.363 (3)		
			
N1—C1—N3	122.0 (2)	H4A—C4—H4C	109.5
N1—C1—C4	120.7 (2)	H4B—C4—H4C	109.5
N3—C1—C4	117.3 (2)	C1—N1—C2	115.85 (19)
N4—C2—N2	119.4 (2)	C2—N2—C3	116.07 (18)
N4—C2—N1	115.07 (19)	C1—N3—C3	119.81 (19)
N2—C2—N1	125.6 (2)	C1—N3—H3	115 (2)
N5—C3—N2	120.5 (2)	C3—N3—H3	124 (2)
N5—C3—N3	118.8 (2)	C2—N4—H4D	120.0
N2—C3—N3	120.7 (2)	C2—N4—H4E	120.0
C1—C4—H4A	109.5	H4D—N4—H4E	120.0
C1—C4—H4B	109.5	C3—N5—H5A	120.0
H4A—C4—H4B	109.5	C3—N5—H5B	120.0
C1—C4—H4C	109.5	H5A—N5—H5B	120.0
			
N3—C1—N1—C2	0.9 (3)	N5—C3—N2—C2	179.7 (2)
C4—C1—N1—C2	−179.6 (2)	N3—C3—N2—C2	−1.3 (3)
N4—C2—N1—C1	−179.9 (2)	N1—C1—N3—C3	−1.6 (4)
N2—C2—N1—C1	−0.3 (3)	C4—C1—N3—C3	178.8 (2)
N4—C2—N2—C3	−179.9 (2)	N5—C3—N3—C1	−179.1 (2)
N1—C2—N2—C3	0.6 (3)	N2—C3—N3—C1	1.9 (3)

### Hydrogen-bond geometry (Å, °)

**Table:**

*D*—H···*A*	*D*—H	H···*A*	*D*···*A*	*D*—H···*A*
N3—H3···Cl1^i^	0.86 (1)	2.25 (1)	3.107 (2)	174 (3)
N4—H4D···Cl1^ii^	0.86	2.52	3.372 (2)	169
N4—H4E···N1^iii^	0.86	2.32	3.171 (3)	170
N5—H5A···N2^ii^	0.86	2.15	3.008 (3)	174
N5—H5B···Cl1	0.86	2.40	3.125 (2)	143

Symmetry codes: (i) −*x*+2, −*y*+1, −*z*+1; (ii) −*x*+1, −*y*+1, −*z*+2; (iii) −*x*, −*y*, −*z*+2.
